# Accelerated fibrin clot degradation is associated with arterial thromboembolism in patients following venous thrombosis: a cohort study

**DOI:** 10.1038/s41598-021-00411-6

**Published:** 2021-10-26

**Authors:** Sandra Mrozinska, Ewa Wypasek, Elżbieta Broniatowska, Anetta Undas

**Affiliations:** 1 Department of Metabolic Diseases, Faculty of Medicine, Jagiellonian University Medical College, Krakow, Poland; 2 Department of Metabolic Diseases, University Hospital, Krakow, Poland; 3 Krakow Centre for Medical Research and Technologies, John Paul II Hospital, Krakow, Poland; 4 Faculty of Medicine and Health Science, Andrzej Frycz Modrzewski Krakow University, Krakow, Poland; 5 Institute of Cardiology, Faculty of Medicine, Jagiellonian University Medical College, 80 Pradnicka St, 31-202 Krakow, Poland

**Keywords:** Predictive markers, Thromboembolism, Thrombosis

## Abstract

Several lines of evidence have suggested that patients following venous thromboembolism (VTE) are at higher risk of arterial thromboembolism (ATE). Prothrombotic fibrin clot characteristics were reported in individuals with cardiovascular risk factors. We investigated whether specific fibrin clot properties measured after 3–4 months of anticoagulation characterize VTE patients with subsequent ATE. We enrolled 320 patients following VTE aged below 70 years (median age, 46). Ten patients were lost to follow-up. ATE occurred in 21 individuals after a median 54 (31–68) months during a follow-up of 87.5 months (incidence 0.94%; 95% confidence interval [CI], 0.59–1.4 per patient-year). Patients with ATE had faster fibrin clot degradation, reflected by maximum rate of D-dimer increase during plasma clot lysis induced by tissue-type plasminogen activator (D-D_rate_) at baseline. Clot permeability, turbidimetric variables, clot lysis time, and thrombin generation were unrelated to ATE. Univariable Cox proportional hazards analysis showed that age, diabetes, and D–D_rate_ were risk factors for subsequent ATE. Increased D–D_rate_ (by 0.001 mg/L/min; hazard ratio, 1.08; 95% CI 1.02–1.14) was an independent predictor of ATE after adjustment for potential confounders. Faster fibrin clot degradation at 3 months since VTE may increase the risk of ATE among VTE patients during follow-up.

## Introduction

With an overall incidence of 1 to 2% of individuals per year, venous thromboembolism (VTE) involving deep-vein thrombosis (DVT) and pulmonary embolism is a common disease and its prevalence increases with age^1^. A two-way relationship between VTE and arterial thromboembolism (ATE), largely manifests as myocardial infarction (MI) and ischemic cerebrovascular events, has been reported, however the available data are inconclusive^2–6^.

Population-based studies have demonstrated that patients with prior unprovoked VTE had a 1.3-fold higher risk of ATE compared to controls during 20 years of follow-up^6^, and patients aged 20–39 years after unprovoked VTE had almost fourfold increased risk of MI in the 10-year follow-up study^7^. Individuals with unprovoked VTE have been found to be at a higher risk of ATE as compared to those with provoked VTE (incidence rate ratio, 1.86; 95% confidence interval [CI], 1.19–2.89) and controls (incidence rate ratio, 1.87; 95%CI, 1.32–2.65)^4^. It has been also reported that patients with unprovoked DVT are more likely to have carotid plaques compared with control subjects (odds ratio, 1.8; 95% CI, 1.1–2.9)^8^. Moreover, a higher mortality rate associated with ATE (standardized incidence ratio 1.28; 95% CI 1.00–1.56) was observed in VTE patients^9^.

The mechanisms underlying association between VTE and ATE are not fully understood^3^. VTE and ATE share some risk factors, including older age, male sex and obesity^2,10,11^. Trauma, immobility and thrombophilia represent well-established VTE risk factors, however thrombophilia is also reported to be associated with ATE^10,12–15^, while cigarette smoking, hyperlipidemia, diabetes, and hypertension are potent risk factors for ATE^12^. Statins and aspirin used in the prevention of ATE have been reported to reduce the risk of VTE, while anticoagulant agents in particular rivaroxaban, decrease the risk of ATE, which provides additional evidence for the role of blood coagulation in ATE^16,17^.

Looking for prothrombotic mechanisms involved in the pathophysiology of both ATE and VTE, it has been demonstrated that fibrin clots generated from plasma of patients with VTE are characterized by the so-called prothrombotic fibrin clot phenotype, defined as formation of denser fibrin clot networks evidenced by low permeability and reduced susceptibility to lysis, which can predict the risk of recurrent VTE^18–21^. Prothrombotic fibrin clot properties were also observed in patients with acute and prior ischemic stroke or MI^22,23^. Of note, cardiovascular risk factors including smoking, diabetes, higher blood pressure, and positive family history have been shown to be associated with prothrombotic fibrin clot characteristics^18,22,24–26^.

Given the available data suggesting a higher risk of ATE in VTE patients^6,7^, we put forward a hypothesis that prothrombotic fibrin clot properties might identify a subset of VTE patients at increased risk of developing ATE during long-term follow-up. We aimed to evaluate the predictive value of a comprehensive set of plasma fibrin clot parameters in terms of the risk of ATE among VTE patients.

## Results

### At baseline

We studied 320 DVT patients (155 men, 48.4%) at a median age of 46 (36–54) years, including 159 (49.7%) with unprovoked VTE. A total of 112 (35%) subjects smoked cigarettes, 96 (30%) individuals had hypertension, 13 (4.1%) suffered from diabetes, 256 (80%) from hypercholesterolemia and 63 (19.7%) from obesity. Heart failure was diagnosed in 10 (3.1%) patients.

### ATE during follow-up

Ten patients (3.1%) were lost to follow-up. The median follow-up period was 87.5 (77–95) months. ATE was diagnosed in 21 (6.8%, 0.9% per year) patients (9 men, 12 women) after a median follow-up of 54 (31–68) months. Fourteen patients (4.5% of the whole group) had MI (including 7 ST-elevated MI and 7 non-ST elevated MI), 6 (1.9%) had ischemic stroke and 1 patient was diagnosed with peripheral arterial disease complicated with acute popliteal artery occlusion. The incidence rate of ATE was 0.94% (95% CI, 0.59–1.4%) per patient-year. The corresponding incidence rates of MI and ischemic stroke were 0.62% (95% CI, 0.36–1.02%) per patient-year and 0.27% (95% CI, 0.11–0.56%) per patient-year (Table 1). Five subjects of the 21 ATE patients (23.8%) were also diagnosed with recurrent VTE. In all of them ATE was observed after recurrent VTE when patients were on anticoagulation.

**Table 1:** Baseline characteristics of patients divided into groups based on the occurrence of venous thromboembolism (VTE) or arterial thromboembolism (ATE) during follow-up.

Variable	All patients (n = 310)	Non-ATE patients (n = 289)	Patients with ATE (n = 21)	*P*	Patients without recurrent VTE and ATE (n = 211)	Patients with recurrent VTE* (n = 83)	Patients with ATE (n = 16)	*P*
Age, years	46 (36–55)	45 (36–54)	51 (48–56)	0.01	45 (36–53)	48 (39–56)	51.5 (48–56.5)	0.02†
Age > 50 years, n (%)	106 (34.2)	95 (32.9)	11 (52.4)	0.09	66 (31.3)	31 (37.3)	9 (56.3)	0.09
Male sex, n (%)	150 (48.4)	141 (48.8)	9 (42.9)	0.66	109 (51.7)	33 (39.8)	8 (50)	0.18
BMI, kg/m^2^	26 (23.6–29.1)	25.9 (23.5–29.1)	26.4 (25.2–28.1)	0.27	25.9 (23.3–29.4)	26.2 (24.2–28.4)	26.2 (25.2–29.6)	0.62
Unprovoked VTE, n (%)	154 (49.7)	143 (49.5)	11 (52.4)	0.82	96 (45.5)	50 (60.2)	8 (50)	0.08
DVT alone, n (%)	239 (77.1)	220 (76.1)	19 (90.5)	0.43	160 (75.8)	64 (77.1)	15 (93.8)	0.3
Family history of VTE, n (%)	50 (16.1)	49 (17)	1 (4.8)	0.22	37 (17.5)	12 (14.5)	1 (6.3)	0.44
Duration of anticoagulation, months	10 (7–12)	10 (7–12)	10 (8–12)	0.25	10 (6–12)	11 (8–12)	10 (8–12)	0.022‡

*BMI* body mass index, *DVT* deep vein thrombosis. Values are given as median (interquartile range), or numbers (percentages). In terms of ATE two patients excluded from the previous analysis were included in the current follow-up study.* Five patients with both recurrent VTE and ATE were included into recurrent VTE group as this event occurred first.

^†^ Indicates statistical difference between patients without both VTE and ATE, and patients with ATE.

^‡^ Indicates statistical difference between patients without both VTE and ATE, and patients with recurrent VTE.

Among 310 studied patients 154 had unprovoked VTE. Those patients had lower fibrinogen compared to patients with provoked VTE (2.9 [2.5–3.6] g/L versus. 3.2 [2.6–4] g/L, p = 0.02). There were no differences in other cardiovascular risk factors between patients with unprovoked and provoked VTE (data not shown). Fibrinogen correlated negatively with permeability coefficient (K_s_) (r = - 0.37), and positively with both absorbance (ΔAbs) (r = 0.61) and clot lysis time (CLT) (r = 0.24) (Supplemental Table 1).

Patients who experienced ATE during follow-up were older by about 6 years and more frequently suffered from diabetes. There were no other intergroup differences in demographics or comorbidities (Table 1, Supplemental Table 2). Regarding routine laboratory investigations patients with ATE during follow-up had 23% lower high-density lipoprotein cholesterol compared to the remainder (1.14 [1.01–1.55] mmol/L versus 1.48 [1.21–1.73] mmol/L; p = 0.009). There were no differences in fibrinogen, D-dimer, and thrombin generation between patients with ATE and the remainder.

When we divided patients into three groups, i.e. patients with recurrent VTE, patients with ATE, and those free of both ATE and recurrent VTE (Table 1), we observed that patients with ATE were older than those from the latter group. Patients with ATE had 26.7% higher low-density lipoprotein cholesterol (3.8 [3.1–4.2] mmol/L versus 3 [2.5–3.7] mmol/L; p = 0.03) along with 15.2% lower peak thrombin compared to patients with recurrent VTE (Supplemental Table 2).

### Fibrin clots and ATE

**Figure 1:**
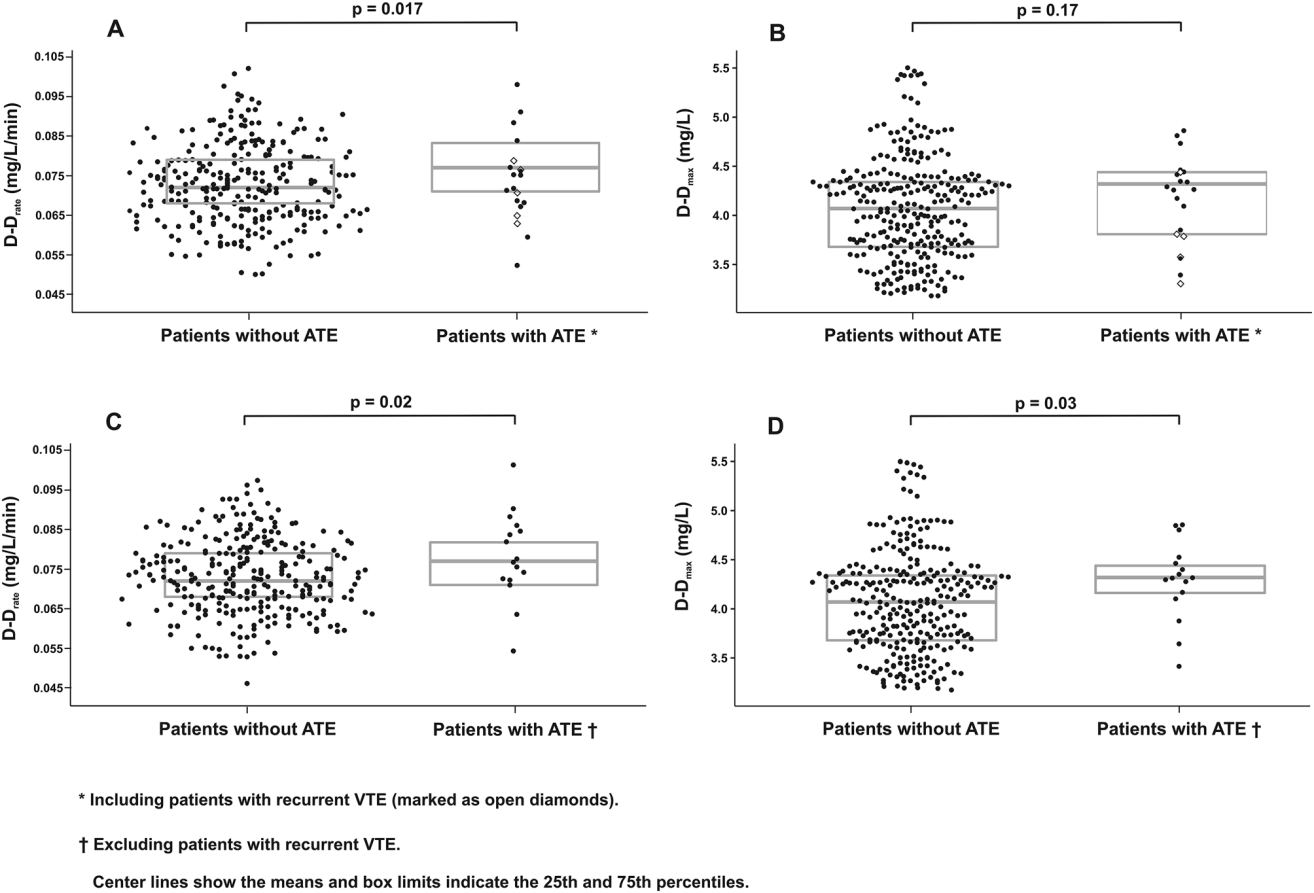
The maximum rate of increase in D-dimer levels (D–D_rate,_ panel (**A**,**C**) and maximum D-dimer concentrations (D–D_max,_ panel **B**,**D**) measured during plasma clot lysis in the permeation assay in patients without arterial thromboembolism (ATE) (n = 289) and patients with subsequent ATE including (n = 21, panel **A**,**B**) and excluding (n = 16, panel **C**,**D**) patients with recurrent VTE (n = 5).

Patients with diagnosed ATE compared with the remainder had baseline lower K_s_ which reflects a smaller average pore size in fibrin networks, as well as a higher rate of increase in D-dimer levels (D-D_rate_) (Table 2, Fig. 1). The two groups did not differ in lag phase, ΔAbs, maximum D-dimer concentrations (D–D_max_), and CLT (Table 2). When 5 subjects with recurrent VTE were excluded from ATE group, then patients with subsequent ATE (n = 16) had higher D-D_rate_ (0.077 [0.071–0.083] mg/L/min versus 0.072 [0.068–0.079] mg/L/min; p = 0.02) and D–D_max_ (4.32 [4.15–4.46] mg/L versus 4.07 [3.68–4.34] mg/L; p = 0.03) (Fig. 1) compared to the remainder (n = 289), without any differences in other fibrin variables, including K_s_.

**Table 2:** The comparison of fibrin clot proprieties.

Variable	All patients (n = 310)	Non-ATE patients (n = 289)	Patients with ATE (n = 21)	*P*	Patients without recurrent VTE and ATE during follow-up (n = 211)	Patients with recurrent VTE*(n = 83)	Patients with ATE (n = 16)	*P*
K_s_, 10^–9^ cm^2^	7.4 (6.5–7.9)	7.4 (6.6–8)	6.8 (6.4–7)	0.036	7.5 (6.9–8.1)	6.6 (6.1–7.3)	7 (6.6–7.5)	< 0.001†
Lag phase, s	43 (39–46)	43 (39–46)	43 (37–46)	0.58	44 (40–47)	40 (35–44)	43.5 (37.5–46)	< 0.001†
ΔAbs	0.81 (0.77–0.85)	0.81 (0.77–0.85)	0.83 (0.8–0.86)	0.09	0.8 (0.76–0.85)	0.83 (0.78–0.87)	0.83 (0.8–0.87)	0.043†
D-D_max_, mg/L	4.07 (3.68–4.39)	4.07 (3.68–4.34)	4.31 (3.8–4.42)	0.17	4.07 (3.64–4.32)	3.98 (3.69–4.41)	4.32 (4.15–4.46)	0.05
D-D_rate_, mg/L/min	0.072 (0.068–0.079)	0.072 (0.068–0.079)	0.077 (0.071–0.083)	0.017	0.072 (0.069–0.08)	0.069 (0.066–0.073)	0.077 (0.071–0.083)	< 0.001† 0.002‡
CLT, min	86 (74–99)	86 (74–99)	82 (70–95)	0.41	81 (71–94)	100 (90–108)	75 (68.5–89)	< 0.001†‡

K_s,_ fibrin clot permeability; ΔAbs, maximum absorbance at the plateau phase; D-D_max,_ maximum D-dimer concentrations; D-D_rate_, rate of increase in D-dimer levels; CLT, clot lysis time; for other abbreviations see Table 1. Values are given as median (interquartile range). In terms of ATE two patients excluded from the previous analysis were included in the current follow-up study.

*Five patients with both recurrent VTE and ATE were included into recurrent VTE group as this event occurred first.

^†^Indicates statistical difference between patients without both VTE and ATE, and patients with recurrent VTE.

^‡^Indicates statistical difference between patients with recurrent VTE and patients with ATE.

The presented differences between groups remained statistically significant after adjustment for age, sex, diabetes and fibrinogen.

**Figure 2:**
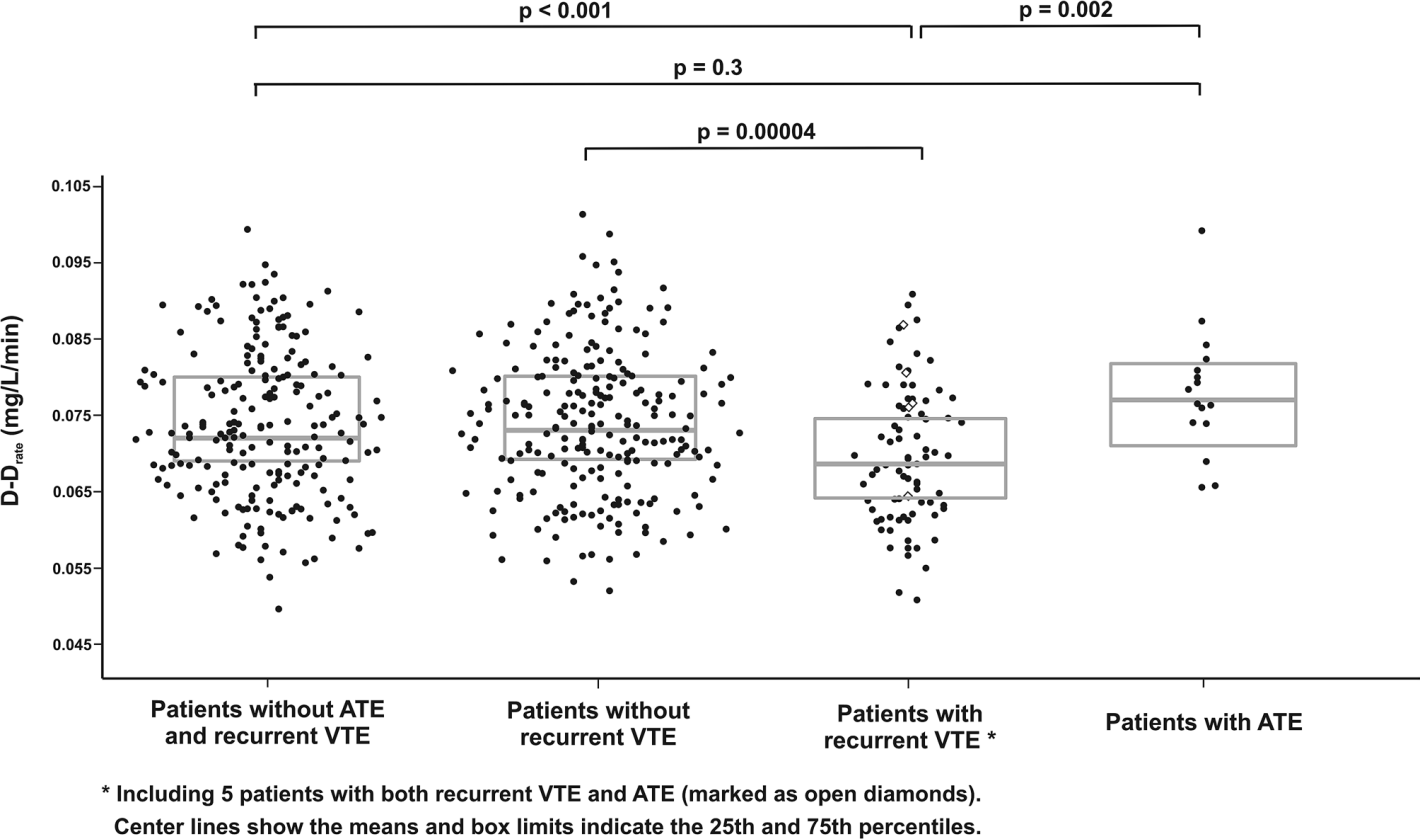
The maximum rate of increase in D-dimer levels (D–D_rate_) for patients without both recurrent venous thromboembolism (VTE) and arterial thrombosis event (ATE), patients without recurrent VTE, patients with recurrent VTE and patients with ATE during follow-up.

We compared patients free of both recurrent VTE and ATE during follow-up with those with recurrent VTE and those with ATE. We found that the latter group had 11.6% higher D-D_rate_ and 25% shorter CLT than those with recurrent VTE (Table 2, Fig. 2).

There were no differences in fibrin clot properties between patients with MI and ischemic stroke (data not shown).

### Risk factors of ATE

Univariable Cox proportional hazards analysis revealed that older age (hazard ratio [HR], 1.05; 95% CI, 1.01–1.1), diabetes (HR, 3.96; 95% CI, 1.17–13.45) and D–D_rate_ (HR, 1.07; 95% CI, 1.014–1.13) predicted ATE during follow-up. Higher D–D_rate_ was a risk factor for ATE after adjustment for age, sex, diabetes, fibrinogen and recurrent VTE (HR, 1.08; 95% CI, 1.02–1.14, Table 3). D–D_rate_ predicted ATE also after adjustment for potential confounders including acetylsalicylic acid (ASA) (HR, 1.08; 95% CI, 1.02–1.14, Supplemental Table 3).

**Table 3:** The univariable and multivariable Cox proportional hazards models for risk factors of ATE.

Variable	HR per	Univariable		Multivariable	
		HR (95% CI)	*P*	HR (95% CI)	*P*
Age	1 year	1.05 (1.01–1.1)	0.02	1.05 (1.005–1.1)	0.03
Male sex	No/Yes	0.81 (0.34–1.91)	0.62	0.68 (0.28–1.65)	0.4
Duration of anticoagulation	1 month	1.07 (0.96–1.19)	0.23		
Unprovoked VTE	No/Yes	1.09 (0.46–2.56)	0.85		
Smoking	No/Yes	1.15 (0.47–2.76)	0.76		
Diabetes	No/Yes	3.96 (1.17–13.45)	0.03	3.75 (1.08–12.98)	0.04
Hypercholesterolemia	No/Yes	1.06 (0.36–3.15)	0.92		
CRP	1 mg/L	0.86 (0.58–1.27)	0.45		
Fibrinogen	1 g/L	1.3 (0.84–2.03)	0.24	1.46 (0.92–2.3)	0.1
D-dimer	100 mg/dL	0.99 (0.64–1.54)	0.98		
ETP	0.001 nM x min	0.58 (0.1–3.21)	0.53		
K_s_	1 × 10^–9^ cm^2^	0.69 (0.45–1.04)	0.08		
Lag phase	1 s	0.98 (0.9–1.07)	0.65		
ΔAbs	0.01	1.06 (0.99–1.13)	0.11		
D-D_max_	1 mg/L	1.45 (0.66–3.17)	0.35		
D-D_rate_	0.001 mg/L/min	1.07 (1.01–1.13)	0.01	1.08 (1.02–1.14)	0.01
CLT	1 min	0.99 (0.97–1.02)	0.63		
Recurrent VTE	No/Yes	0.81 (0.3–2.2)	0.68	0.04 (0.35–3.09)	0.94

*HR* hazard ratio, *CI* confidence interval, *CRP* C-reactive protein, *ETP* endogenous thrombin potential, for other abbreviations see Tables 1 and Table 2.

In terms of ATE two patients excluded from the previous analysis were included in the current follow-up study.

Twenty-one patients with ATE and 289 patients without ATE were included in the Cox proportional hazards models.

Age, gender, diabetes, fibrinogen, D-D rate and recurrent VTE were included in the multivariable model.

C-statistic = 0.73.

### Fibrin clot and recurrent VTE

Individuals with recurrent VTE (n = 83) had 12% lower K_s_, 9.1% lower lag phase and 23.5% longer CLT compared to subjects without both recurrent VTE and ATE during follow-up (Table 2, Fig. 2). Patients with recurrent VTE compared with those free of recurrent VTE, regardless ATE differed significantly in fibrin clot properties (Supplemental Table 4). The results correspond to the findings obtained after a median 44 months of follow-up^20^. Previously, seventy-seven (25%) subjects diagnosed previously with recurrent VTE were characterized by 12% lower K_s,_ 9% shorter lag phase in the fibrin formation assay and 25% longer CLT^20^.

## Discussion

The present study is the first to assess a comprehensive set of plasma fibrin clot properties in a cohort of VTE patients as potential risk factors for ATE in order to address the hypothesis of the fibrin-related mechanisms linking VTE and ATE. Contrary to our expectations, we demonstrated that in a largely middle-aged cohort of VTE patients an increased rate of plasma fibrin clot degradation expressed as higher D-D_rate_ characterized patients with subsequent ATE^27^. The study showed that other fibrin clot properties, including clot permeability and CLT extensively explored in VTE and cardiovascular disease, did not identify young and middle-aged VTE patients at an increased risk of ATE in the future. Our findings suggest that faster enzymatic fibrin clot degradation could reflect the instability of fibrin networks prone to fragmentation in vivo and could increase the risk of ATE. This study appears to indicate that the prothrombotic clot phenotype observed in young and middle-aged subjects after 3 months following VTE might be associated with reduced risk of ATE in the following years. The current observations suggest the complex role of fibrin clot structure and function in the pathophysiology of thromboembolism.

The reported incidence rate of ATE in VTE patients varied between studies from 0.3% per patient year for acute MI in individuals with unprovoked DVT or pulmonary embolism (mean age 46 years) to 3.8% per patient-year for ATE (MI, stroke) in patients with unprovoked pulmonary embolism at a mean age of 55 ± 17 years^4^. The systematic review showed that the weighted mean incidence rate of ATE was 0.65% (95% CI, 0.36–1.01; I^2^ = 25%) per patient-year in randomized controlled trials and 0.76% (95% CI, 0.6–0.94, I^2^ = 96%) per patient-year in cohort studies^4^. We observed a similar incidence rate of ATE, i.e. 0.94% (95% CI, 0.59–1.4%) per patient-year. In the study of Spencer et al.^7^ who studied 6065 young and middle-aged patients (range 20–64 years) after unprovoked VTE, the incidence rate of acute MI was 0.33% per patient-year, which is in line with our findings (0.62% [95% CI, 0.36–1.02%] per patient-year).

Of paramount importance is the plasma-based assay in which we were able to show faster clot lysis in patients who developed ATE during follow-up. In the assay introduced by Collet et al.^28^ in 1999 to analyze fibrin properties in nephrotic patients and healthy controls, the previously formed plasma clot was perfused with a buffer containing high tissue-type plasminogen activator (tPA) concentration, similar to that observed in patients treated with tPA-based thrombolysis^29^. Our modification of the original approach was used for the first time to assess the impact of statins and other drugs on fibrin clot properties in 2006^30^. This assay was also applied by our group to evaluate efficiency of fibrinolysis in patients with ATE. We found higher D–D_rate_ and D–D_max_ in patients with cryptogenic ischemic stroke as compared to the control group^23^. In contrast, lower D-D_rate_ was observed in MI patients who survived in-stent thrombosis^31^ and patients with a history of limb ischemia of unknown cause as compared to the control group^32^. In line with the present study, this measure of clot lysis did not predict recurrent VTE^20^. Our present observation suggests that various lysis assays should be used to highlight specific fibrin clot abnormalities in a particular disease with lysis abnormalities, without any single test appropriate to all conditions in which fibrinolysis is disturbed as shown in previous studies^32–34^. In our study the modified assay by Collet et al.^28^ enabled to show subtle changes in fibrin clot lysability that could be of importance in ATE prediction, while other commonly used assays did not differentiate subjects with VTE at risk of ATE. Importantly we did not observe any differences in CLT between patients who developed the subsequent ATE and the remainder.

Mechanisms behind the observed link between ATE and faster clot degradation remain unclear. Atherogenic effects of fibrin degradation products have been demonstrated in several studies. Corban et al.^35^ have reported that fibrin degradation products were associated with larger atherosclerotic plaques and necrotic core areas. They suggested that higher fibrin degradation products might be a marker of subclinical rupture or erosion of the plaque^35^. Moreover, fibrin was found to stimulate the production of proinflammatory molecules, interleukin-1, interleukin-8 and intracellular adhesion molecule-1^36–38^ Fibrin degradation products stimulate the migration of monocytes and local fibrinolysis^39^.

On the other hand, the role of fibrin in atherothrombosis is complex. It has been suggested that fibrinogen and fibrin are involved in the development of early atherosclerotic lesions and their progression^39–41^. In hyperlipidemic and hypercoagulable mouse models (mice carrying the factor V Leiden and mice being thrombomodulin mutants) Seehaus et al.^27^ observed that hypercoagulabity leaded to larger atherosclerotic plaques and plaque stability with less necrotic cores, and that anticoagulant treatment reduced plaque stability^27^. Borissoff et al.^42^ reported enhanced procoagulant state and higher endogenous thrombin potential in the early atherosclerotic lesions as compared to stable advanced atherosclerotic lesions. They concluded that blood coagulation and the resultant increased formation of fibrin contribute to a more stable atherosclerotic plaques^42^. In animal studies it has been shown that in mice less prothrombotic phenotype was associated with reduced atherosclerosis or less early-stage atherosclerotic lesions^43^. Based on the current findings, it might be speculated that faster lysis with enhanced fragmentation of fibrin meshworks can predispose to embolic events. Moreover, faster clot degradation could be associated with more vulnerable plaques with more fragile fibrin deposits on the surface of atherosclerotic lesions, and therefore it could be a risk factor of ATE. It remains to be established whether similar observation can be made in all subjects at risk of ATE beyond those with a history of VTE episode.

In our cohort there were no differences in the risk of ATE between patients with unprovoked VTE versus provoked VTE. This finding, though based on a relatively small number of patients, was contrary to our expectations, because several studies, including its meta-analysis, have shown that patients after unprovoked VTE were at higher risk of ATE compared to those with provoked VTE, however some reports failed to observe such differences related to the type of VTE^4,6,44^. Of note, we observed an increased fibrinogen concentration at 3 months since the event in patients with provoked VTE. Elevated fibrinogen levels are observed commonly in patients with cardiovascular disease and show associated with cardiovascular risk factors^45,46^. This parameter is also the key determinant of fibrin clot properties^18^.

Several study limitations should be acknowledged. The study population was limited, however this cohort was well described with a large set of hemostatic parameters and followed for, on average, more than 7 years. All laboratory measurements were done in a single point time, at 3 months and this time point is of key importance in clinical decision making following VTE. Changes over time in all the variables measured cannot be ruled out, however in our opinion the impact of the results obtained specifically after 3 months of anticoagulation has been shown suggesting persistent abnormalities affecting clinical outcomes during follow-up^19,23^. The influence of drugs including ASA on fibrin clot properties is possible^18^. We did not perform microscopic assessment of plasma clots, but in our previous studies D-D_rate_ showed no association with fiber diameter or pore size in clots obtained using scanning electron microscopy. The current findings cannot be easily extrapolated to elderly VTE patients in whom ATE and VTE occur more commonly, since we excluded such patients from this study. The same holds true for anticoagulated VTE patients because the majority of the current cohort stopped anticoagulation after a few months of treatment except those who developed recurrent VTE episodes.

In conclusion, we demonstrated that patients with ATE which is experienced a few years since VTE are not characterized by the prothrombotic fibrin clot phenotype in a cohort of patients aged 70 years or less, which is in contrast to the association between such phenotype and recurrent VTE. The finding suggesting that VTE patients with subsequent ATE during follow-up had a higher rate of clot degradation at high concentrations of rtPA, measured in vitro after 3 months of anticoagulation supports growing evidence for fibrin involvement in a wide spectrum of thromboembolic episodes. It needs further investigations whether D-D_rate_ determined at 3 months since the index VTE may help identify VTE patients who need close surveillance for ATE and assessment of cardiovascular risk. Further studies should be performed to corroborate our results in larger cohorts and elucidate pathophysiological mechanisms behind the contribution of fibrin degradation and the clinical outcomes.

## Patients and methods

A total of 368 patients with a history of VTE were screened for meeting the eligibility criteria for the study between October 2008 and June 2010. The cohort was described in detail in our previous paper^20^. Briefly, the eligible patients following the first-ever isolated DVT or combined with pulmonary embolism were recruited among those referred to our center for diagnostic work-up. The diagnosis of DVT and pulmonary embolism was established as reported^20^. We excluded patients with known cancer, severe thrombophilia, recent acute ATE, acute infection or severe kidney or liver failure. Patients with VTE received standard anticoagulant treatment with vitamin K antagonists for 3 to 12 months according to the guidelines^47^.

At enrolment data on cardiovascular risk factors were collected. Hypertension was regarded as increased blood pressure (systolic blood pressure ≥ 140 mmHg or diastolic blood pressure ≥ 90 mmHg) or antihypertensive treatment. Hypercholesterolemia was defined as a total cholesterol level above 5 mmol/l, low-density lipoprotein cholesterol above 3 mmol/l or statin treatment; diabetes as fasting glucose of ≥ 7.0 mmol/l, non-fasting glucose ≥ 11.1 mmol/l, antidiabetic treatment or previously diagnosed diabetes. Obesity was defined as body mass index equal to or greater than 30 kg/m^2^. Heart failure was recognized based on typical symptoms and reduced left ventricular ejection fraction (< 40%).

All experimental protocols were approved by the Bioethics Committee of the Jagiellonian University. From all study participants the informed written consent in accordance with the Declaration of Helsinki was obtained. All methods were carried out in accordance with relevant guidelines and regulations.

### Follow-up

Patients were followed-up on a 6-month basis since enrolment. Clinical data was collected every six months via a visit in the outpatient clinic or phone calls.

The primary composite endpoint was ATE defined as ischemic stroke, MI or peripheral arterial thromboembolic event. Stroke was diagnosed based on persistence of typical symptoms for more than 24 h confirmed on magnetic resonance imaging or computed tomography^48^. The diagnosis of MI was established based on typical symptoms, changes on the electrocardiogram and increased myocardial necrosis biomarkers^49^. Peripheral arterial thromboembolic event was defined as acute peripheral artery occlusion^50^. ATE episodes related to invasive procedures were excluded. The secondary endpoint of the study was symptomatic VTE diagnosed based on positive findings of color duplex sonography. Recurrent DVT in the same leg as the index event was diagnosed when new no-compressibility of venous segment was observed or when there was an increase of at least 4 mm in the residual diameter. Anticoagulation treatment was prescribed again in patients with recurrent VTE.

### Laboratory investigations

After 3 months (12 to 15 weeks) of anticoagulant treatment since DVT, antecubital blood samples for laboratory investigation were taken from fasting patients in the morning hours (8 to 10 AM). Patients who were treated with vitamin K antagonists were first temporarily switched to a low-molecular-weight heparin for 10–14 days. Blood samples were taken after 16–24 h since the last injection. We assessed blood cell count, lipid profiles, glucose, creatinine, and international normalized ratio using routine laboratory techniques. Firstly, blood samples (vol/vol, 9:1 of 3.2% trisodium citrate) were centrifuged at 2000 × *g* for 10 min within 30 min of the draw, then we removed supernatant, aliquoted and stored it at − 80 °C until analysis. We determined fibrinogen using the Clauss method. High-sensitivity C-reactive protein was assessed by nephelometry (Siemens). We measured plasma concentrations of D-dimer, tPA and plasminogen activator inhibitor-1 antigens using immunoenzymatic assays (American Diagnostica). Thrombophilia screening was conducted as described^51^.

### Fibrin clot permeability

Fibrin clot permeation was assessed as described^30^. Briefly, we mixed 60 µl of plasma with 60 µl of the coagulation trigger containing 1 IU/ml human thrombin and 20 mM CaCl_2_. Immediately, 100 µl of prepared assay was transferred to a plastic cylinder made from a serological pipette (Sarstedt, Nümbrecht, Germany). Subsequently, after 2 h of incubation at a room temperature, we connected tubes containing the clots via plastic tubing to a reservoir of a buffer (0.05 M tris–HCl, 0.15 NaCl, pH 7.5). The volume of a buffer flowing through the gels was measured within 60 min. Bromophenol blue was used after experiments to find potential leaks, thanks to this procedure we were able to detect and discard the defective clots. We calculated the permeability coefficient, an indirect measure of the average pore size in the fiber network, using the equation: K_s_ (× 10^–9^ cm^2^) = Q × L × η/t × A × ∆P, where Q (cm^3^) is the flow rate at time t (s), L (cm) is the length of the fibrin gel, η (dyne × s/cm^2^) is the viscosity of the liquid, A (cm^2^) is the cross-sectional area and ∆p (dyne/cm^2^) is differential pressure. The intraassay coefficient of variation was 6.8%.

### Turbidity measurements

To initiate polymerization plasma citrated samples from each patient were mixed 2:1 with a Tris buffer containing 0.6 IU/mL human thrombin (Sigma) and 50 mM CaCl_2_. We used spectrophotometer to read absorbance (ΔAbs) at 405 nm. A lag phase as time to the start of fibrin polymerization, slope of the polymerization curve, along with maximum absorbance at plateau were assessed^19^.

### Plasma clot lysis assays

Fibrinolysis efficiency was examined using two assays at 2 various concentrations of recombinant tPA (rtPA)^30,52^. In the first assay, CLT was assessed. As previously described^20^, 100 µl citrated plasma was mixed with 15 mmol/L CaCl_2_, 0.6 pM human tissue factor (Innovin, Siemens), 12 µmol/L phospholipid vesicles and 60 ng mL^-1^ tPA (rtPA, Boehringer Ingelheim, Ingelheim, Germany). The mixture was transferred to a microtiter plate. Absorbance was measured at 405 nm at 37 °C. CLT was determined as the time from the midpoint of the clear-to-maximum-turbid transition (clot formation), to the midpoint of the maximum-turbid-to-clear transition (the lysis of the clot).

In the second assay, fibrin clot lysis was assessed using a dynamic lysis assay according to Collet et al.^28^ with some modifications^30^. Fibrin clots obtained in the same manner as for fibrin clot permeation were washed with Tris buffer and perfused with the same buffer containing 0.2 µmol/L rtPA (Boehringer Ingelheim). The lysis rate was determined by fibrin degradation reflected D-dimer levels in the effluent using ELISA (American Diagnostica). The D-dimer level was assessing at 20 min intervals until when the fibrin gel collapsed under the pressure, usually after 80–120 min. We measured the maximum rate of increase in D-dimer levels (D–D_rate_) and maximum D-dimer concentrations (D–D_max_) as previously described^20^.

### Calibrated automated thrombogram (CAT)

The commercial reagents (Thrombinoscope, BV, Maastricht, Netherlands) were used to conduct the CAT assay^19^. Shortly, 20 µl of a starting reagent containing 5 pM recombinant relipidated TF, 4 mM phospholipids, 100 mM CaCl_2_ and 2.5 mM fluorogenic substrate was added to 80 µl of plasma sample. We determined the 3 following variables: the peak thrombin, the endogenous thrombin potential, and the time to thrombin using the Fluoroskan Ascent microplate fluorometer (Thermo Fisher Scientific, Vantaa, Finland).

### Statistical analysis

Continuous variables were presented as mean ± standard deviation or median (interquartile range). The Shapiro–Wilk test was used to verify the assumption of the normal distribution of continuous variables. The Student’s or the Welch’s t-test based on the equality of variances for normally distributed variables were used to compare two groups. The Mann–Whitney *U*-test was performed to compare two groups for non-normally distributed continuous variables. The categorical (qualitative) variables were presented as the number (percentages) and the Chi-squared test (or Fisher exact test) was used to compare them between groups and the post-hoc test was applied where it was necessary. To compare three groups the Kruskal–Wallis test was used and the post-hoc comparisons for the Kruskal–Wallis test were also performed.

The risk factors of ATE were revealed using univariable and multivariable Cox proportional hazards regression models. The variables for multivariable Cox regression model were chosen based on p-value less than 0.1 of univariable models (with the exception of Ks) and potential confounders were considered. We included in the final multivariable model: age, gender, diabetes, fibrinogen D–D_rate_ and recurrent VTE. The proportional hazard assumption was verified by the Schoenfeld Residuals test. The predictive accuracy of presented model was estimated by C-index (c-statistics), also referred to as area under Receiver Operating Curve. The goodness of fit for the presented model was assessed by the Grønnesby and Borgan test and also with application of deviance residuals. The results of all Cox regression models are showed as HRs with 95% CIs. The level of significance for the two-sided tests was set below 0.05. The study was powered to have 81% chance of detecting deference in D–D_rate_, given the type I error 5%. Hence, type II error was 19%. The package *R*^53^, G*Power v. 3.1.9.4^54^ and Statistica 12.5 software (StatSoft Inc., Tulsa, Oklahoma, United States) were used to conduct the analyses*.*

## Supplementary Information

Supplementary Information.
