# Supplementary material for: Accelerated fibrin clot degradation is associated with arterial thromboembolism in patients following venous thrombosis: a cohort study
Source: Sci Rep. 2021 Oct 26;11:21003. doi: 10.1038/s41598-021-00411-6 (PMC8548328; doi:10.1038/s41598-021-00411-6)
Supplement: Supplementary file 1 — Supplementary Information. [file 41598_2021_411_MOESM1_ESM.docx]

**Supplemental Table 1. The correlations between fibrinogen, D-dimer, thrombin generation and fibrin clot properties (n = 310).**

| Variable | Fibrinogen, g/L | D-dimer, mg/dL | Peak thromin, nM | ETP, nM×min | Time to thrombin peak, min | K_s_,  10^-9^cm^2^ | Lag phase, s | ΔAbs | D-D_max_, mg/L | D-D_rate_, mg/L/min | CLT, min |
| --- | --- | --- | --- | --- | --- | --- | --- | --- | --- | --- | --- |
| Fibrinogen, g/L | - | 0.1 | -0.07 | 0.21* | -0.14* | -0.37* | -0.05 | 0.61* | 0.14* | -0.15* | 0.24* |
| D-dimer, mg/dL | 0.1 | - | 0.13* | 0.14* | 0.02 | -0.18* | -0.11 | 0.19* | 0.01 | -0.04 | 0.24* |
| Peak thromin, nM | -0.07 | 0.13* | - | 0.37* | -0.25* | -0.16* | -0.12* | 0.06 | -0.01 | -0.008 | 0.22* |
| ETP, nM×min | 0.21* | 0.14* | 0.37* | - | -0.36* | -0.49* | -0.17* | 0.33* | 0.01 | -0.0001 | 0.33* |
| Time to thrombin peak, min | -0.14* | 0.02 | -0.25* | -0.36* | - | 0.09 | -0.01 | -0.14* | -0.06 | -0.07 | -0.14* |
| K_s_, 10^-9^cm^2^ | -0.37* | -0.18* | -0.16* | -0.49* | 0.09 | - | 0.35* | -0.42* | -0.18* | 0.13* | -0.53* |
| Lag phase, s | -0.05 | -0.11 | -0.12* | -0.17* | -0.01 | 0.35* | - | -0.23* | -0.28* | 0.06 | -0.33* |
| ΔAbs | 0.61* | 0.19* | 0.06 | 0.33* | -0.14* | -0.42* | -0.23* | - | 0.25* | -0.01 | 0.39* |
| D-D_max_, mg/L | 0.14* | 0.01 | -0.01 | 0.01 | -0.06 | -0.18* | -0.28* | 0.25* | - | -0.01 | 0.24* |
| D-D_rate_, mg/L/min | -0.15* | -0.04 | -0.008 | -0.0001 | -0.07 | 0.13* | 0.06 | -0.1 | -0.01 | - | -0.13* |
| CLT, min | 0.24* | 0.24* | 0.22* | 0.33* | -0.14* | -0.53* | -0.33* | 0.39* | 0.24* | -0.13* | - |

ETP, endogenous thrombin potential, K_s,_ fibrin clot permeability; ΔAbs, maximum absorbance at the plateau phase; D-D_max,_ maximum D-dimer concentrations; D-D_rate_, rate of increase in D-dimer levels; CLT, clot lysis time.

*p<0.05

**Supplemental Table 2. Additional comparisons of patients divided into groups based on the occurrence of venous thromboembolism (VTE) or arterial thromboembolism (ATE) during follow-up.**

| **Variable** | **All patients**  **(n=310)** | **Non-ATE patients**  **(n=289)** | **Patients with ATE**  **(n=21)** | ***P*** | **Patients without recurrent VTE and ATE**  **(n=211)** | **Patients with recurrent VTE***  **(n=83)** | **Patients with ATE**  **(n=16)** | ***P*** |
| --- | --- | --- | --- | --- | --- | --- | --- | --- |
| **Cardiovascular risk factors, n (%)** |  |  |  |  |  |  |  |  |
| Cigarette smoking | 108  (34.8) | 100  (34.6) | 8  (38.1) | 0.81 | 83  (39.3) | 18  (21.7) | 7  (43.8) | 0.004† |
| Smoking > 10 cigarettes per day | 11  (3.5) | 11  (3.8) | 0  (0) | 0.99 | 1  (0.5) | 10  (12.0) | 0  (0) | < 0.0001† |
| Hypertension | 96  (31) | 92  (31.8) | 4  (19) | 0.33 | 71  (33.6) | 22  (26.5) | 3  (18.8) | 0.27 |
| Diabetes | 13  (4.2) | 10  (3.5) | 3  (14.3) | 0.049 | 7  (3.3) | 4  (4.8) | 2  (12.5) | 0.2 |
| Hypercholesterolemia | 250  (80.6) | 233  (80.6) | 17  (81) | 0.99 | 174  (82.5) | 62  (74.7) | 14  (87.5) | 0.25 |
| Heart failure | 10  (3.2) | 9  (3.1) | 1  (4.8) | 0.51 | 7  (3.3) | 2  (2.4) | 1  (6.3) | 0.72 |
| Obesity | 63  (20.3) | 58  (20.1) | 5  (23.8) | 0.78 | 45  (21.3) | 14  (16.9) | 4  (25) | 0.62 |
| **Medications, n (%)** |  |  |  |  |  |  |  |  |
| Aspirin | 45  (14.5) | 41  (14.2) | 4  (19) | 0.52 | 38  (18) | 4  (4.8) | 3  (18.8) | 0.003† |
| ACEI | 52  (16.8) | 49  (17) | 3  (14.3) | 0.99 | 41  (19.4) | 8  (9.6) | 3  (18.8) | 0.13 |
| β-blockers | 11  (3.5) | 10  (3.5) | 1  (4.8) | 0.54 | 7  (3.3) | 3  (3.6) | 1  (6.3) | 0.83 |
| **Laboratory parameters** |  |  |  |  |  |  |  |  |
| Creatinine, µmol/L | 70  (61.9-79.6) | 70  (61.9-79.6) | 66  (60-76) | 0.23 | 69.8  (61.9-79.6) | 70.7  (62-80) | 68  (60.5-75.5) | 0.42 |
| CRP, mg/L | 1.5  (0.9-2.3) | 1.5  (0.9-2.3) | 1.1  (0.8-2) | 0.2 | 1.5  (0.9-2.3) | 1.7  (1.1-2.2) | 1.1  (0.8-2.9) | 0.19 |
| INR | 0.98  (0.9-1.03) | 0.97  (0.9-1.03) | 1.02  (0.91-1.04) | 0.25 | 0.97  (0.89-1.03) | 0.98  (0.91-1.04) | 1.01  (0.91-1.04) | 0.44 |
| Fibrinogen, g/L | 3  (2.5-3.9) | 3  (2.5-3.9) | 3.6  (3.1-3.8) | 0.13 | 3.1  (2.6-3.9) | 2.9  (2.5-3.6) | 3.4  (2.9-3.8) | 0.2 |
| D-dimer, mg/dL | 277  (223-337) | 275  (226-337) | 293  (218-318) | 0.92 | 258  (215-310) | 315  (250-429) | 292  (218-314.5) | <0.001† |
| tPA antigen, ng/mL | 9.6  (7.2-11.5) | 9.6  (7.2-11.5) | 10.2  (9-11.2) | 0.49 | 9.6  (7.6-11.2) | 9.3  (6.8-11.9) | 10.5  (9.2-11.4) | 0.5 |
| PAI-1, ng/mL | 11.2  (8.5-14.2) | 11.2  (8.5-13.9) | 11.9  (8.1-16.7) | 0.46 | 11.2  (8.5-13.5) | 11.2  (8.4-16.7) | 13.3  (8.6-16.9) | 0.37 |
| Peak thrombin, nM | 247.2  (211-294.8) | 248.2  (210-295) | 246.2  (216-285) | 0.94 | 239  (202-279.5) | 286  (237-349.6) | 242.5  (213.5-267.6) | <0.001†  0.02 ‡ |
| ETP, nM x min | 1535.2  (1356.4-1672) | 1535  (1343-1674) | 1535.4  (1396-1585) | 0.73 | 1510  (1302-1644) | 1617.2  (1421-1783) | 1525.7  (1378.6-1653.5) | 0.0007† |
| Time to thrombin peak, min | 4.67  (4.21-5.33) | 4.67  (4.23-5.33) | 4.94  (4.04-6.0) | 0.7 | 4.7  (4.2-5.4) | 4.8  (4.2-5.3) | 4.9  (4.4-6) | 0.34 |
| **Genotyping, n (%)** |  |  |  |  |  |  |  |  |
| Factor V Leiden | 40  (12.9) | 38  (13.1) | 2  (9.5) | 0.99 | 30  (14.2) | 8  (9.6) | 2  (12.5) | 0.57 |
| Prothrombin 20210A | 15  (4.8) | 15  (5.2) | 0  (0) | 0.61 | 11  (5.2) | 4  (4.8) | 0  (0) | 0.64 |

ACEI, angiotensin-converting enzyme inhibitor; CRP, C-reactive protein; INR, international normalized ratio; tPa, tissue plasminogen activator; PAI-1, plasminogen activator inhibitor-1; for other abbreviations see Supplemental Table 1.

Values are given as median (interquartile range), or numbers (percentages).

In terms of ATE two patients excluded from the previous analysis were included in the current follow-up study.

* Five patients with both recurrent VTE and ATE were included into recurrent VTE group as this event occurred first.

† Indicates statistically significant difference between patients without both VTE and ATE, and patients with recurrent VTE.

‡ Indicates statistically significant difference between patients with recurrent VTE and patients with ATE.

**Supplemental Table 3. Multivariable Cox proportional hazards model for risk factors of ATE including acetylsalicylic acid (ASA) use.**

| **Variable** | **HR per** | **Multivariable** | |
| --- | --- | --- | --- |
|  |  | **HR (95% CI)** | ***P*** |
| Age | 1 year | 1.05 (1.005-1.1) | 0.03 |
| Male sex | No/Yes | 0.68 (0.28-1.64) | 0.39 |
| Diabetes | No/Yes | 3.64 (0.99-13.37) | 0.05 |
| ASA use | No/Yes | 1.09 (0.33-3.54) | 0.89 |
| Fibrinogen | 1 g/L | 1.45 (0.92-2.3) | 0.11 |
| D-D_rate_ | 0.001 mg/L/min | 1.08 (1.02-1.14) | 0.007 |

HR, hazard ratio; CI, confidence interval; for other abbreviations see Supplemental Table 1 and Supplemental Table 2.

In terms of ATE two patients excluded from the previous analysis were included in the current follow-up study.

C-statistic=0.73.

**Supplemental Table 4.** The comparison of fibrin clot properties between patients with recurrent VTE and patients free of recurrent VTE during a median follow up of 87.5 (77-95) months.

| **Variable** | **Patients with recurrent VTE (n=83)** | **Patients without recurrent VTE (n=227)** | ***P*** |
| --- | --- | --- | --- |
| K_s_, 10^-9^cm^2^ | 6.6 (6.1-7.3) | 7.5 (6.9-8.1) | <0.001 |
| Lag phase, s | 40 (35-44) | 44 (40-47) | <0.001 |
| ΔAbs | 0.83 (0.78-0.87) | 0.8 (0.77-0.85) | 0.02 |
| D-D_max_, mg/L | 3.98 (3.69-4.41) | 4.07 (3.66-4.33) | 0.39 |
| D-D_rate_, mg/L/min | 0.069 (0.066-0.073) | 0.073 (0.069-0.08) | 0.001 |
| CLT, min | 100 (90-108) | 80 (70-94) | 0.001 |

For abbreviations see Supplemental Table 1 and Supplemental Table 2.

The presented differences between groups remained statistically significant after adjustment for age, sex, diabetes and fibrinogen.
